# DASHR: database of small human noncoding RNAs

**DOI:** 10.1093/nar/gkv1188

**Published:** 2015-11-08

**Authors:** Yuk Yee Leung, Pavel P. Kuksa, Alexandre Amlie-Wolf, Otto Valladares, Lyle H. Ungar, Sampath Kannan, Brian D. Gregory, Li-San Wang

**Affiliations:** 1 Department of Pathology and Laboratory Medicine, University of Pennsylvania, Philadelphia, PA 19104, USA; 2 Penn Institute for Biomedical Informatics, University of Pennsylvania, Philadelphia, PA 19104, USA; 3 Genomics and Computational Biology Graduate Group, University of Pennsylvania, Philadelphia, PA 19104, USA; 4 Department of Computer and Information Science, University of Pennsylvania, Philadelphia, PA 19104, USA; 5 Department of Biology, University of Pennsylvania, Philadelphia, PA 19104, USA; 6 Institute on Aging, University of Pennsylvania, Philadelphia, PA 19104, USA

## Abstract

Small non-coding RNAs (sncRNAs) are highly abundant RNAs, typically <100 nucleotides long, that act as key regulators of diverse cellular processes. Although thousands of sncRNA genes are known to exist in the human genome, no single database provides searchable, unified annotation, and expression information for full sncRNA transcripts and mature RNA products derived from these larger RNAs. Here, we present the *Database of small human noncoding RNAs (DASHR)*. DASHR contains the most comprehensive information to date on human sncRNA genes and mature sncRNA products. DASHR provides a simple user interface for researchers to view sequence and secondary structure, compare expression levels, and evidence of specific processing across all sncRNA genes and mature sncRNA products in various human tissues. DASHR annotation and expression data covers all major classes of sncRNAs including microRNAs (miRNAs), Piwi-interacting (piRNAs), small nuclear, nucleolar, cytoplasmic (sn-, sno-, scRNAs, respectively), transfer (tRNAs), and ribosomal RNAs (rRNAs). Currently, DASHR (v1.0) integrates 187 smRNA high-throughput sequencing (smRNA-seq) datasets with over 2.5 billion reads and annotation data from multiple public sources. DASHR contains annotations for ∼48 000 human sncRNA genes and mature sncRNA products, 82% of which are expressed in one or more of the curated tissues. DASHR is available at http://lisanwanglab.org/DASHR.

## INTRODUCTION

In recent years, there has been a tremendous growth of interest in studying different kinds of small (<100 nucleotides (nt)) non-coding RNAs (sncRNAs) (1). This is because many classes of sncRNAs, which are transcribed from noncoding genomic regions (2), regulate various aspects of gene expression during normal animal physiology and development. These sncRNAs control gene expression through regulation of chromatin architecture, transcription, as well as RNA splicing, editing, translation and turnover (3,4). Some better studied sncRNA classes include microRNAs (miRNAs), which exhibit diverse expression patterns and important functional roles in many cellular processes (5). Also, PIWI-interacting RNAs (piRNAs) are involved in transposon silencing (6), epigenetic programming, DNA rearrangements, mRNA turnover and translational control (7). Both small nuclear and nucleolar RNA (snRNAs and snoRNAs) are essential in splicing and ribosomal RNA processing (4), and participate in tumorigenesis (8). The small cytoplasmic RNAs (scRNAs) or Y-RNAs are involved in DNA replication (9) and alternative splicing (10). Additionally, transfer RNAs (tRNAs) not only play a central role in translation (11), but are also involved in stress signaling. Besides, mutations in tRNAs can lead to complex human diseases (12). Finally, ribosomal RNAs (rRNAs) are the cellular machinery for protein translation and synthesis.

Currently, the biological roles and molecular functions of many mature sncRNA products in human are still unexplored. Recent advances in small RNA sequencing (smRNA-seq) technology (13) and the development of numerous computational tools (14,15) allow for the *in vivo* and *in vitro* characterization of sncRNA genes and their mature products in a genome-wide manner. This not only enables us to identify new members of well-known functional classes such as miRNAs more efficiently, but also leads to the discovery of new classes of sncRNAs such as tRNA fragments (tRFs), which are derived from sequences directly upstream or downstream of mature tRNAs (16) and have been shown to play multiple roles in cellular physiology (17).

Though smRNA-seq has great potential for identifying and characterizing the various classes of sncRNAs simultaneously, it is nonetheless challenging to study sncRNA gene expression based on smRNA-seq data due to the lack of comprehensive and unified annotation across all major classes of sncRNAs. This is because most non-coding RNA databases only focus on one specific type of sncRNA gene and/or precursor sncRNA, i.e. full, unprocessed sncRNA transcripts. These include the most prominent databases such as miRBase (18) for miRNAs, snoRNA-LBME-db (19) for snoRNAs, and tRNAdb (20) for tRNAs. Moreover, the use of different experimental data for quantifying expression levels of precursor RNAs across each of these databases makes it challenging to explore multiple classes of sncRNAs simultaneously. In addition, no single database provides truly comprehensive information for integrated annotation, expression, and processing information for human sncRNA genes and their derived mature sncRNA products.

To address this gap, we have developed DASHR, a database of genome-wide small human noncoding RNAs. The goal of DASHR is to serve as a unified catalog of annotation, sequence, structural and expression information for human sncRNA genes and their mature products. In fact, DASHR is the first attempt to systematically integrate the annotation, sequence, RNA secondary structure, expression, and evidence for specific processing for eight major classes of sncRNA genes and their corresponding mature sncRNA products across 42 normal human tissues and cell types. To accomplish this, the annotation data was compiled and integrated from many public sources. Additionally, expression data was produced by uniformly analyzing 187 manually collected and curated high-throughput sequencing (smRNA-seq) datasets (see Materials and Methods). Thus, the current release of DASHR (v1.0) contains over 48,000 precursor and mature sncRNA records, 82% of which have experimental expression data that support their expression in one or more human tissues. Altogether, DASHR will aid the broader scientific community in exploring the genomic landscape of human sncRNA abundance and processing, with data that can be directly compared.

## DATABASE CONTENTS

DASHR is substantially more comprehensive than existing non-coding RNA databases (Supplementary Table S1) as it integrates a greater set of human sncRNA classes and their annotations with a significantly larger number of curated high-throughput smRNA-seq datasets (over 180 smRNA-seq libraries corresponding to ∼2.5B total reads, Supplementary Table S2).

**Figure 1.**
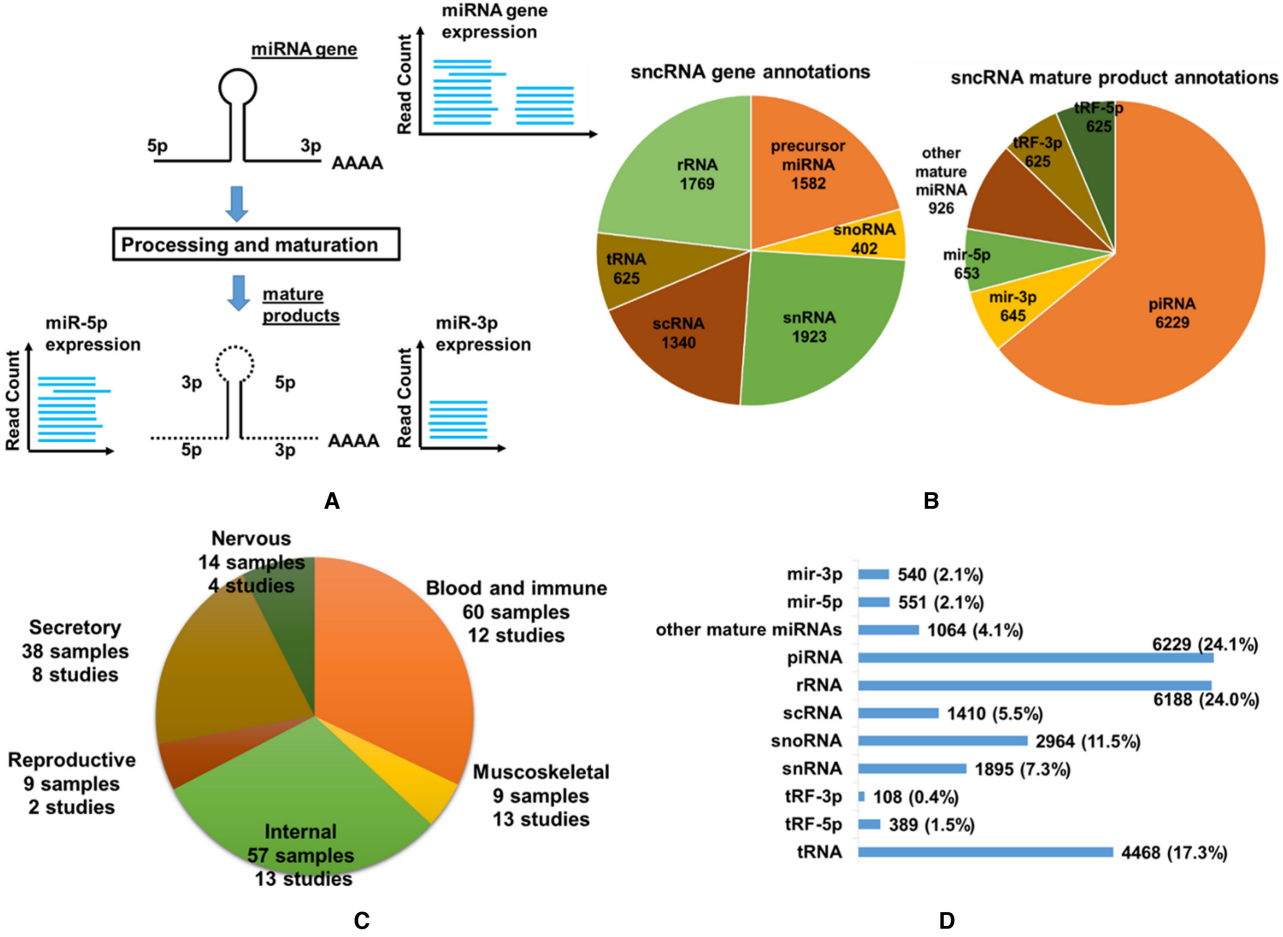
(**A**) Schematic diagram showing the relationship between a sncRNA gene and its mature products using miRNA gene as an example. (**B**) Number of annotations for sncRNA genes (left) and mature sncRNA products (right) in DASHR. (**C**) Number of samples and studies present for each human body system in DASHR. In total, DASHR contains over 180 high-throughput smRNA-seq datasets with over 2.5 billion processed reads. (**D**) Annotated mature sncRNA products with expression levels in DASHR.

DASHR contains information on both sncRNA gene and mature RNA products (the relationship between gene and mature products is illustrated in Figure 1a). The current version of sncRNA annotation available through DASHR (v1.0, July 2015) contains 7,641 sncRNA gene records (precursor miRNAs, rRNAs, scRNAs, snRNAs, snoRNAs, tRNAs) and 9703 annotated mature sncRNA product records (i.e. mature miRNAs, piRNAs, tRFs, etc.) (Figure 1b), corresponding to 48 075 genomic loci. The curated and processed smRNA-seq data (summarized in Figure 1c) overlapped with these sncRNA annotations provides expression profiles for 6301 (82%) annotated sncRNA genes that can be queried in DASHR. This corresponds to 25 086 expressed mature sncRNA products (Figure 1d) across all tissues (8881/9703 of original mature sncRNA records together with 16 925 mature products derived from 7641 sncRNA gene records).

To validate the expression data in DASHR, we have compared the levels of miRNAs reported by DASHR from the brain prefrontal cortex to the benchmark miRNA expression data from brain (microarray) that was reported in (21). We observed significant overlap between miRNAs with expression above the median (76.3% [45/59 miRNAs]) and significant correlation between expression levels in DASHR and the benchmark dataset (Spearman rank correlation *ρ* = 0.5119 (−1 ≤ *ρ* ≤1), *P*-value = 2.7476e−09) for the full miRNA panel (119 assayed miRNAs). This level of correlation is typical for comparisons across technologies (microarray versus RNA sequencing, see, e.g. (22)) as well as across tissues (prefrontal cortex versus hippocampus).

The annotated sncRNAs in DASHR derive from a diverse array of genomic elements including promoters (7%), UTRs (6%), exons (15%), introns (6%) and intergenic regions (66%) (Supplementary Figure S1). The number of mature sncRNA products in DASHR per tissue and cell type is shown in (Supplementary Figure S2). Supplementary Table S2 summarizes all smRNA-seq datasets that have been incorporated into DASHR.

## FEATURES OF THE DATABASE

DASHR allows researchers to study sncRNA expression as well as their respective processing patterns. DASHR aims to provide a simple and unified resource to the scientific community allowing users to (i) query the expression and processing information for sncRNA genes and mature sncRNA products of interest *in vivo* across human tissues and cell types in normal, non-diseased states; (ii) browse sncRNAs and mature sncRNA products for specific human tissues or cell types and (iii) retrieve annotations across various sncRNA classes simultaneously for any genomic interval.

### Search

DASHR is available online at http://lisanwanglab.org/DASHR. Users can locate records in the database by any of these three types of queries:

1. list records by the full or partial name of sncRNA, its HGNC symbol, RefSeq ID, UCSC ID, or name of the gene containing the sncRNA;
2. locate RNA loci overlapping with given genomic coordinates based on the hg19 reference genome annotation
3. query by raw RNA sequence.

DASHR will return a list of matching entries and an ID conversion table containing the corresponding sncRNA names, the HGNC symbols, RefSeq IDs, and UCSC IDs. Users can view the genomic and expression information of a specific sncRNA entry by clicking on the sncRNA name in the tables under ‘View DASHR annotation’.

### Summary of annotation and expression information

**Figure 2.**
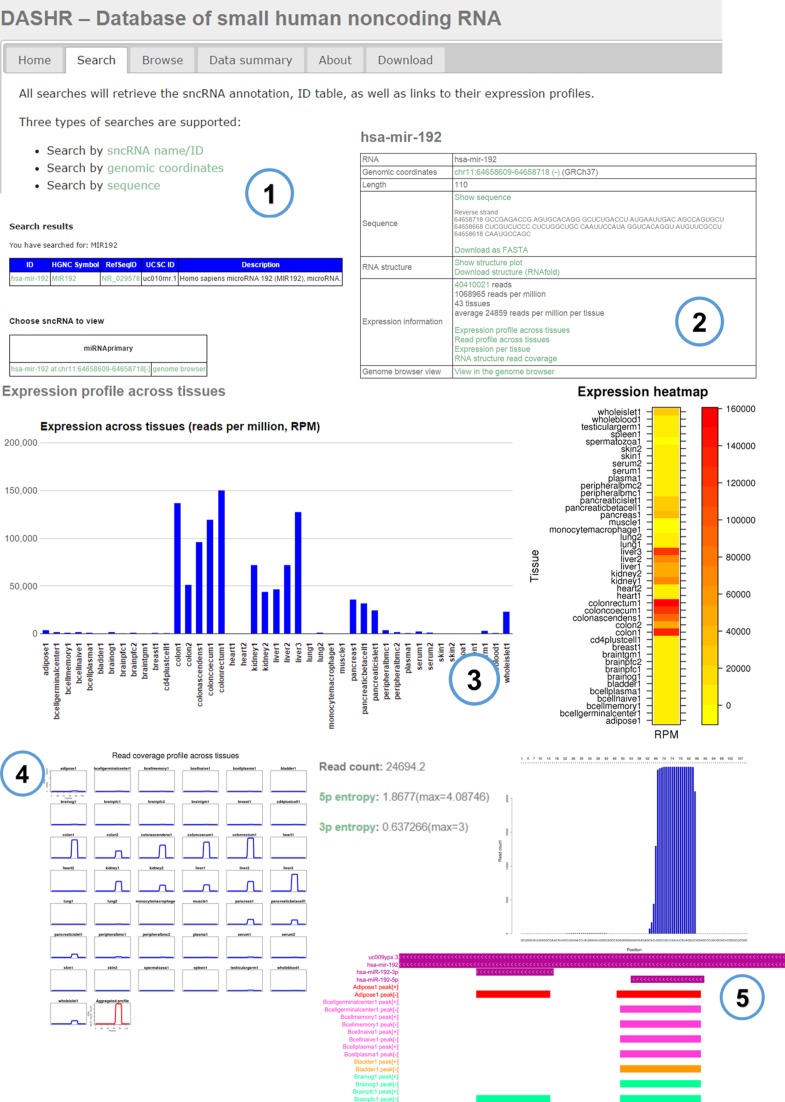
A DASHR entry web page for a sncRNA search (**1**) showing the four sections: (**2**) a summary table contains both annotation and summary of expression information; (**3**) expression profile across tissues; (**4**) read coverage profile across tissues or per tissue; (**5**) genome browser for mapped sequences.

The resulting ‘View DASHR annotation’ page displays expression and genomic information for sncRNAs as shown in Figure 2.

Specifically, the summary table (Panel 2 in Figure 2) displays the annotation information, including the sequence of the selected locus, genomic coordinates, structural information (RNAfold (23) predicted secondary structure) and the length of the RNA locus. This table also includes summary statistics for the sncRNA's expression information, highlighting the total number of experiments with observed expression, total raw read count, and total read counts in reads per million (RPM). Moreover, the table links to a UCSC genome browser view of the locus with mapped sequencing data across all tissues and cell types.

The expression information (Panels 3–5 in Figure 2) for sncRNA is represented by (1) a summary expression profile across tissues (in bar plot or heat map format); (2) the read coverage profile plot across individual tissues, as well as an aggregated profile, which is the sum of profiles across all tissues; (3) the read coverage plot per tissue; (4) a figure of sequencing read coverage plotted against the RNAfold-predicted secondary structure produced by SAVoR (24) and (5) a genome browser view of the mapped sncRNA data for all tissues and cell types.

All these plots and expression tables can be downloaded from the same page.

### sncRNA processing information

In addition to quantifying the expression profiles across tissues for each sncRNA entry, we also include the processing specificities for both ends of every sncRNA product for each tissue using cleavage specificity scores (calculated using CoRAL (14)). The specificity of RNA cleavage is estimated using entropy measurements. This information is available in the tissue-specific section of the sncRNA entry page along with the read coverage profile for the sncRNA locus. The entropy measurement is computed based on the distributions of the 5p and 3p read end positions of all sncRNA reads mapped to the locus. This feature is designed to distinguish between random, non-specific RNA products and RNA products processed by specific RNA cleaving enzymes. The entropy measurement captures the specificity (or degeneracy) of RNA cleaving enzymes specific to the production of different types of sncRNAs. For example, the processing of mature miRNA products from precursor miRNAs tends to have a stable 5p cleavage position (resulting in lower values of the entropy measurement) and more variable 3p ends (higher entropy) (14).

## DATABASE DESIGN AND ORGANIZATION

**Figure 3.**
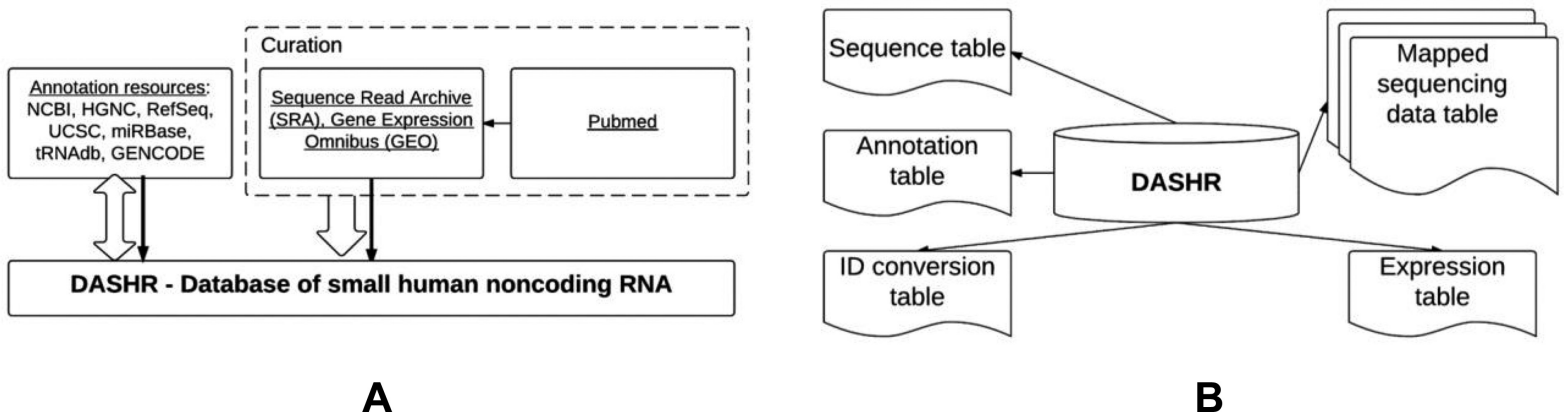
(**A**) Overview of DASHR. Block arrows indicate data flow, while solid arrows indicate web traffic. (**B**) Organization of DASHR. Data tables are connected by lines indicating they are directly associated with DASHR.

DASHR has been implemented as an easily updatable and modular architecture. Figure 3 gives the overview of the DASHR database system. The contents of DASHR are derived from integration of many annotation resources as well as curation and processing of publicly available smRNA sequencing datasets. First, the annotation resources from existing databases are collected and compiled into a unified catalog (Figure 3A). Second, the publicly available smRNA-seq datasets are curated, processed, and imported into DASHR on a regular basis (Figure 3B).

Internally, the information in DASHR is organized into tables (Figure 3B). Specifically, the sequence table, annotation table, and ID conversion table contain basic information for each unprocessed and mature sncRNA entry. Each sncRNA entry is uniquely identified by a DASHR identifier, which serves as the primary key for all tables in DASHR. The mapped sequencing data tables are the tables generated from smRNA-seq data after performing processing and quality control steps (see Materials and Methods). They contain genome-wide continuous valued expression data in track format. The expression table contains expression values for each sncRNA entry in DASHR, which is obtained after segmentation, quantification, and annotation (see Materials and Methods). For more detailed information on the database schema and implementation, refer to ‘DASHR implementation’ on the ‘About’ page in our website.

## MATERIALS AND METHODS

DASHR reports the annotation, RNA secondary structure, expression, and evidence of specific RNA processing (cleavage specificity scores/entropy) of sncRNA genes, precursors and mature sncRNA products across different human tissues and cell types. Refer to Supplementary Figure S3 for the workflow in DASHR.

### Annotation resources

DASHR integrates multiple existing annotation resources. The annotation information for miRNAs is based on miRBase (v19) (18); snRNA, snoRNA, scRNA and rRNA annotations are from UCSC genome browser (25) and GENCODE (26); tRNA information is based on tRNAdb (20); and piRNA annotation is derived from NCBI (27). The current annotation in DASHR also includes tRNA fragment (tRF) annotations that are created based on 5p and 3p 50 nt sequences upstream and downstream of the known tRNA genes. Information from NCBI (27), HGNC (28), RefSeq (29), and UCSC (25) were used to build the ID conversion table for each processed and mature sncRNA entry in DASHR. All of these annotations are based on the GRCh37/hg19 human reference genome, which is currently still broadly used. We also plan to upgrade DASHR in the near future to GRCh38/hg38 once more compatible RNA annotations become available.

### Data collection and curation of smRNA-seq

We manually curated Illumina smRNA-seq datasets from GEO (30) and SRA (31). These datasets were obtained from sequencing non-diseased human tissue samples and cell types for studying or profiling sncRNAs. The full list of included samples and smRNA-seq libraries is shown in Supplementary Table S2.

### Categorizing into tissue and cell types

We manually categorized the smRNA-seq samples into different groups of tissues and cell types, stratified by experimental sources (refer to Study ID found in Supplementary Table S2).

### Processing smRNA-seq datasets

After curation and categorization, we used a modified version of our previously developed pipeline CoRAL (14) to standardize the processing of smRNA-seq datasets and generate sncRNA expression levels. The pipeline can be summarized into three parts.

#### Processing and quality control

We first identified the correct adapter sequence (Supplementary Table S2, Illumina adapter column) and trimmed the sequencing reads using cutadapt (32). We then mapped the trimmed reads to a standardized version of the human reference genome (GRCh37/hg19). The reads were aligned using STAR (33) allowing for multi-mapping. Over 93% of the trimmed reads were mapped to the human genome on average per dataset (Supplementary Table S2 details trimming and alignment statistics for each smRNA-seq dataset).

#### Segmentation and quantification

Previous tools to identify transcriptionally active regions or read blocks (34–36) do not identify peaks with evidence of specific processing patterns, i.e. mature RNA products (e.g. low 5p read entropy). To fit our goal of identifying mature sncRNA products and quantifying their expression levels in addition to precursors, we developed a customized approach to identify peaks with evidence of specific processing for mature sncRNA products at base pair resolution. To do this, we scanned the genomic sequence and identified the start of the peak by finding two adjacent positions with at least a 2-fold increase in the number of mapped reads. Similarly, the corresponding end of the peak is found by looking for at least a 2-fold decrease in the number of mapped reads. Additionally, the detected peaks need to have at least 10 reads. After identifying the mature sncRNA locations, we then quantified the number of reads falling within these regions as read counts for each sncRNA. To enable comparison across tissues, we took into account the library size information for each of the sequencing experiments and reported the read count in ‘reads per million’ (RPM), since this is the most commonly used normalization method to account for differences in library size across different experiments (37).

#### Annotation of mature RNA products

We overlapped the peaks with evidence of specific processing (identified in the previous step) with DASHR annotation (see Annotation resources). Each peak is assigned to its precursor RNA class and sncRNA gene.

## DATA AVAILABILITY

Currently, users can download all the resources in DASHR from the ‘Download’ page. These include:

1. annotations for all sncRNA entries and mature products in DASHR;
2. sequences of all sncRNA entries in DASHR;
3. a sncRNA ID conversion table which contains cross-referenced IDs for each sncRNA entry;
4. an expression table summarizing the expression in reads per million (RPM) across all tissues in DASHR;
5. an expression table summarizing the expression in raw read counts (RAW) across all tissues in DASHR;
6. an annotation and expression table summarizing mature sncRNA products expressed in each tissue;
7. a file summarizing the detailed information of all smRNA-seq samples/datasets curated and analyzed for inclusion in DASHR; and
8. all the mapped sequencing data for each tissue.

## FUTURE DEVELOPMENTS

The current release (v1.0) of DASHR is from July 2015. DASHR is designed with the prospects of future expansion in mind, and we plan to continuously increase the data available through this database by curating and processing new sequencing datasets generated from any human tissues or cell types in non-diseased conditions. Additionally, future functionalities to be integrated will include information on RNA secondary structure, target interactions, RNA modifications, as well as providing differential expression and tissue specificity information.

## CONCLUSIONS

DASHR is the first large-scale effort to unify annotation resources, structural, expression, and processing information for genes and mature products from eight prominent classes of sncRNAs. In addition, using the curated and processed smRNA-seq data, we provide not only the basic annotation information for each sncRNA entry in DASHR, but also expression levels and processing specificities for each entry across 42 different human tissues or cell types in normal, non-diseased conditions. Thus, DASHR is distinct from other existing databases because it integrates annotations for all major classes of sncRNAs with baseline expression profiles in different human tissues and cell types, making it a very useful resource to the broader scientific community. DASHR is freely available for use at http://lisanwanglab.org/DASHR.
